# Osmotic therapies added to antibiotics for acute bacterial meningitis

**DOI:** 10.1002/14651858.CD008806.pub3

**Published:** 2018-02-06

**Authors:** Emma CB Wall, Katherine MB Ajdukiewicz, Hanna Bergman, Robert S Heyderman, Paul Garner

**Affiliations:** University College LondonDivision of Infection and ImmunityGower StreetLondonUKWC1E 6BT; Pennine Acute Hospitals NHS TrustDepartment of Infectious DiseasesNorth Manchester General HospitalDelaunays Road, CrumpsallManchesterUKMB 5RB; CochraneCochrane ResponseSt Albans House57‐59 HaymarketLondonUKSW1Y 4QX; University of Malawi College of MedicineMalawi‐Liverpool‐Wellcome Clinical Research ProgrammeP. O Box 30096BlantyreChichiriMalawi; Liverpool School of Tropical MedicineDepartment of Clinical SciencesPembroke PlaceLiverpoolMerseysideUKL3 5QA

## Abstract

**Background:**

Every day children and adults die from acute community‐acquired bacterial meningitis, particularly in low‐income countries, and survivors risk deafness, epilepsy and neurological disabilities. Osmotic therapies may attract extra‐vascular fluid and reduce cerebral oedema, and thus reduce death and improve neurological outcomes.

This is an update of a Cochrane Review first published in 2013.

**Objectives:**

To evaluate the effects of osmotic therapies added to antibiotics for acute bacterial meningitis in children and adults on mortality, deafness and neurological disability.

**Search methods:**

We searched CENTRAL (2017, Issue 1), MEDLINE (1950 to 17 February 2017), Embase (1974 to 17 February 2017), CINAHL (1981 to 17 February 2017), LILACS (1982 to 17 February 2017) and registers of ongoing clinical trials (ClinicalTrials.com, WHO ICTRP) (21 February 2017). We also searched conference abstracts and contacted researchers in the field (up to 12 December 2015).

**Selection criteria:**

Randomised controlled trials testing any osmotic therapy in adults or children with acute bacterial meningitis.

**Data collection and analysis:**

Two review authors independently screened the search results and selected trials for inclusion. Results are presented using risk ratios (RR) and 95% confidence intervals (CI) and grouped according to whether the participants received steroids or not. We used the GRADE approach to assess the certainty of the evidence.

**Main results:**

We included five trials with 1451 participants. Four trials evaluated glycerol against placebo, and one evaluated glycerol against 50% dextrose; in addition three trials evaluated dexamethasone and one trial evaluated acetaminophen (paracetamol) in a factorial design. Stratified analysis shows no effect modification with steroids; we present aggregate effect estimates.

Compared to placebo, glycerol probably has little or no effect on death in people with bacterial meningitis (RR 1.08, 95% CI 0.90 to 1.30; 5 studies, 1272 participants; *moderate‐certainty evidence*), but may reduce neurological disability (RR 0.73, 95% CI 0.53 to 1.00; 5 studies, 1270 participants; *low‐certainty evidence*).

Glycerol may have little or no effect on seizures during treatment for meningitis (RR 1.08, 95% CI 0.90 to 1.30; 4 studies, 1090 participants; *low‐certainty evidence*).

Glycerol may reduce the risk of subsequent deafness (RR 0.64, 95% CI 0.44 to 0.93; 5 studies, 922 participants; *low to moderate‐certainty evidence*).

Glycerol probably has little or no effect on gastrointestinal bleeding (RR 0.93, 95% CI 0.39 to 2.19; 3 studies, 607 participants; *moderate‐certainty evidence*). The evidence on nausea, vomiting and diarrhoea is uncertain (RR 1.09, 95% CI 0.81 to 1.47; 2 studies, 851 participants; *very low‐certainty evidence*).

**Authors' conclusions:**

Glycerol was the only osmotic therapy evaluated, and data from trials to date have not demonstrated an effect on death. Glycerol may reduce neurological deficiency and deafness.

## Plain language summary

**Osmotic therapies added to antibiotics for acute bacterial meningitis**

**What is the aim of this review?**

The aim of this Cochrane Review is to collect and analyse trials evaluating osmotic therapies given orally or intravenously to people with acute bacterial meningitis. Cochrane authors collected and analysed all relevant studies to answer this question; they found five relevant studies.

**Key messages**

Giving glycerol, an osmotic diuretic, probably has little or no effect on death (*moderate‐certainty evidence*), but may reduce subsequent deafness (*moderate‐certainty evidence)* or neurological disability (*low‐certainty evidence*). The evidence is current to 17 February 2017.

**What was studied in the review?**

In meningitis, the cerebrospinal fluid that surrounds the brain and spinal cord is infected, usually as a result of spread from the blood. Any form of meningitis can result in death or severe disability, but acute bacterial meningitis is rapidly fatal without treatment. Even with antibiotics, 10% to 15% of children with bacterial meningitis die in high‐income countries with much higher rates in low‐income settings. The infection causes the brain to swell, and this is thought to contribute to death and to long‐term brain damage in survivors. Osmotic therapies increase the concentration of the blood by exerting an osmotic pressure across a semi‐permeable membrane (such as a cell wall or blood vessel lining in the brain). This draws water from the brain into the blood, thereby reducing pressure in the brain. Potentially osmotic therapies could increase the rate of survival, or they could do harm.

**What are the main results of the review?**

We included five trials that compared glycerol with placebo in a total of 1451 patients with bacterial meningitis. In the studies steroids were often given as well, but this did not appear to modify any of the effects seen with glycerol.

This review detected no benefit from glycerol relating to death. There appeared to be marginal protection against deafness and against neurological disability. No effect on epileptic seizures at follow‐up was noted. Glycerol was not associated with any severe adverse effects. The number of trials included was small and only two tested a large number of participants. All trials were from different healthcare settings and examined either adults or children.

## Summary of findings

**Summary of findings for the main comparison:** Glycerol for acute bacterial meningitis

**Glycerol for acute bacterial meningitis**						
**Patient or population:** children and adults with acute bacterial meningitis **Settings:** Finland, India, South America, Malawi **Intervention:** glycerol with or without steroids compared with placebo. All participants received broad‐spectrum antibiotics						
**Outcomes**	Illustrative comparative risks* (95% CI)		**Relative effect (95% CI)**	**Number of participants (studies)**	**Quality of the evidence (GRADE)**	**Comments**
	**Assumed risk**	**Corresponding risk**				
	**Control**	**Glycerol**				
**Death**	**19 per 100**	**21 per 100** (17 to 25)	**RR 1.08** (0.90 to 1.30)	1272 (5 studies)	⊕⊕⊕⊝ M**oderate**^1,2,3,4^	Downgraded for imprecision. Glycerol probably has little or no effect on death
**Neurological disability**	**9 per 100**	**6 per 100** (5 to 9)	**RR 0.73** (0.53 to 1.00)	1270 (5 studies)	⊕⊕⊝⊝ **Low**^1,3,4,5^	Downgraded for imprecision and inconsistency. Glycerol may reduce disability
**Seizures**	**32 per 100**	**35 per 100** (29 to 42)	**RR 1.08** (0.90 to 1.30)	1090 (4 studies)	⊕⊕⊝⊝ **Low**^1,3,4,6^	Downgraded for inconsistency and imprecision. Glycerol may have little or no effect on seizures
**Hearing loss**	**16 per 100**	**10 per 100** (7 to 15)	**RR 0.64** (0.44 to 0.93)	922 (5 studies)	⊕⊕⊕⊝ **Moderate**^1,2,3,7^	Downgraded for imprecision. Glycerol probably reduces hearing loss
**Adverse effects: nausea, vomiting, diarrhoea**	**47 per 100**	**51 per 100** (38 to 69)	**RR 1.09** (0.81 to 1.47)	851 (2 studies)	⊕⊝⊝⊝ **Very low**^1,3,4,8,9^	Downgraded for serious inconsistency and imprecision. The effect of glycerol on adverse events: nausea, vomiting and diarrhoea is uncertain
**Adverse effects: gastrointestinal bleeding**	**3 per 100**	**3 per 100** (13 to 8)	**RR 0.93** (0.39 to 2.19)	607 (3 studies)	⊕⊕⊕⊝ **Moderate**^1,2,3,4^	Downgraded for imprecision. Glycerol probably has little or no effect on adverse events: gastrointestinal bleeding
*The basis for the **assumed risk** (e.g. the median control group risk across studies) is provided in footnotes. The **corresponding risk** (and its 95% confidence interval (CI)) is based on the assumed risk in the comparison group and the **relative effect** of the intervention (and its 95% CI). **CI:** confidence interval **RR:** risk ratio						
GRADE Working Group grades of evidence **High quality:** Further research is very unlikely to change our confidence in the estimate of effect **Moderate quality:** Further research is likely to have an important impact on our confidence in the estimate of effect and may change the estimate **Low quality:** Further research is very likely to have an important impact on our confidence in the estimate of effect and is likely to change the estimate **Very low quality:** We are very uncertain about the estimate						

^1^No serious risk of bias: allocation concealment was adequate in four trials and unclear (not reported) in one trial. ^2^Not downgraded for inconsistency. ^3^Not downgraded for indirectness. The five trials were conducted in Finland, Malawi, India and South America. Four were in children and one in adults. All included patients with suspected meningitis and cerebrospinal fluid (CSF) changes suggestive of bacterial infection. ^4^Downgraded by one level for imprecision: the 95% CI includes what might be a clinically important harm and no effect with glycerol. ^5^Downgraded by one level for inconsistency: in the Finnish trial the risk of neurological sequelae was reduced with glycerol (RR 0.50, 95% CI 0.32 to 0.78, N = 329), but this was not found in the other studies and the meta‐analysis did not detect a difference (I² = 59%). ^6^Downgraded by one level for inconsistency: in the trial with adults the risk of seizures was higher with glycerol (RR 1.62, 95% CI 1.18 to 2.23, N = 250), but this was not found in the other studies and the meta‐analysis did not detect a difference (I² = 62%). ^7^Downgraded by one level for imprecision: the number of patients with reported hearing loss was low in these studies and the 95% CI includes both no effect and what might be a clinically important benefit with glycerol. Larger studies would be necessary to have full confidence in this effect. ^8^Another two trials reported on this outcome but the results could not be added to the meta‐analysis; one reported more cases of vomiting with glycerol and the other that the incidence of vomiting was "similar" in the treatment groups. ^9^Downgraded by two levels for inconsistency: in the South American and Finnish trials the risk of adverse effects was increased with glycerol, but this was not found in the Malawi and India trials, and the meta‐analysis did not detect a difference (I² = 79%).

## Background

### Description of the condition

Community‐acquired acute bacterial meningitis is a devastating infection with associated rates of death and disability that have changed little over the last 10 to 15 years. In high‐income countries, 5% to 30% of adult patients die, rising to 50% to 60% in low‐income countries, despite highly effective antibiotics against the causative pathogens (de Gans 2002; Nguyen 2007; Scarborough 2007). The high mortality is predominately seen in *Streptococcus pneumoniae* (*S pneumoniae*) infections; meningitis caused by *Neisseria meningitidis* (*N meningitidis*) carries a lower mortality. In children, a wider range of pathogens are noted and the case fatality rate is lower (Harnden 2006; Molyneux 2006; Pelkonen 2009; Peltola 2009; Roine 2009). Nevertheless, some survivors develop neurological problems that may be permanent. The most common meningitis sequelae are deafness, epilepsy and poor cognitive development (Molyneux 2002; Nguyen 2007; van de Beek 2009), thought to be caused by infection‐induced inflammation, thrombosis and brain oedema (swelling). The outcome from bacterial meningitis is influenced by the pathogen, the geographical area, the patient's access to healthcare and the quality of the healthcare system. There are very few data on risk factors for poor outcomes in low‐income countries. However, anaemia and delayed presentation to hospital are probably important (McCormick 2012; Sudarsanam 2017). HIV may influence outcomes but the role of the virus in pathogenesis is not yet clearly understood (Domingo 2009). High mortality rates, despite effective antibiotics, have led investigators to try and minimise neurological inflammation with adjunctive therapies.

Increasing understanding of the pathways of cerebral inflammation in meningitis has led several investigators to try treatments that aim to reduce brain oedema and inflammation and improve brain perfusion. The intervention most extensively tested in clinical trials has been corticosteroids. A Cochrane Review shows a mortality benefit in adults in Europe with meningitis due to *S pneumoniae* and an overall reduction in deafness in adults and children (Brouwer 2015). Another systematic review of individual patient data from five randomised studies suggests that the effect of dexamethasone on outcomes for bacterial meningitis in these countries is limited to reducing the incidence of hearing loss in survivors (van de Beek 2010). A long‐held concern exists over excessive fluids contributing to brain oedema; a further Cochrane Review suggests that judicious fluid resuscitation guided by the clinical condition is appropriate to maximise brain perfusion without contributing to brain oedema (Maconochie 2016).

### Description of the intervention

Osmotic therapies work by increasing the concentration of the blood. They exert an osmotic pressure across a semi‐permeable membrane (such as a cell wall or blood vessel lining in the brain), which draws water from the brain into the blood and reduces pressure in the brain. This is theoretically advantageous if brain swelling is causing reduction in brain function.

Osmotic therapies have long been used in acute brain trauma (BTF 2000), and their use has been postulated in other forms of acute brain injury, particularly stroke (Bereczki 2007; Yu 1992; Yu 1993) and cerebral malaria (Namutangula 2007; Okoromah 2011). Mannitol and hypertonic saline are the most commonly used osmotic therapies (Wakai 2013), but glycerol, sorbitol and sodium lactate have also been investigated (Righetti 2004; Stoll 1998). Details of all these therapies are reported in Table 2. Glycerol has been studied in animals with meningitis, where no effect was noted. Conclusions from these studies are limited by the applicability of animal models of meningitis, where set doses of pathogenic bacteria are introduced directly into the animal's central nervous system, to the complex host pathogen interactions in human disease (Blaser 2010; Schmidt 1998). The excellent safety profile of glycerol in previous studies (Righetti 2004), combined with its low cost and easy administration and availability, has led investigators to look for its efficacy as an adjuvant treatment in acute bacterial meningitis in both adults and children, particularly in low‐income countries.

**1:** Available osmotic therapies

**Drug**	**Class**	**Mechanism of action**	**Dose range and route**	**Studied/used in**
Glycerol	Sugar alcohol	Probably osmosis plus possible vascular and metabolic benefit	IV 5% to 10% solution or 50 g Oral 1.5 g/kg	Meningitis (Peltola 2007), stroke (Righetti 2004)
Mannitol	Sugar alcohol	Osmotic diuretic	IV 20% solution 1 mL/kg to 10 mL/kg or 1 g/kg	Brain trauma (Wakai 2013), cerebral malaria (Namutangula 2007), stroke (Bereczki 2007)
Sorbitol	Sugar alcohol	Osmotic diuretic (weak)	Oral, IV	Experimental brain perfusion, stroke
Hypertonic saline	Hypertonic solutions	Osmosis	IV	Brain trauma (Choi 2005), stroke (Schwarz 2002)
Sodium lactate	Hydroxy acids	Osmosis (weak)	IV	Brain trauma (Ichai 2009)

IV: intravenous

### How the intervention might work

All osmotic therapies have slightly different and poorly understood mechanisms of action. The osmotic drug's mechanism of action causes dehydration of central nervous system (CNS) cells, lowering intracranial pressure (ICP). However this effect may only be temporary and lead to a rebound phenomenon where cells subsequently draw in too much water, increasing the oedema. Mannitol has this mechanism of action but acts primarily by erythrocyte deformity through increases in intravascular water, allowing increased tissue oxygenation in the CNS. Mannitol produces a large diuresis through this effect, which causes a reflex cerebral vasoconstriction, temporarily reducing ICP. However, there is a significant risk of subsequent rebound raised ICP and mannitol is now used sparingly due to this concern. The main mechanism of action of glycerol in humans is unknown but there are some data to suggest that the addition of glycerol in meningitis could potentially improve cerebral blood flow and metabolism (Mathew 1972; Meyer 1972). Glycerol also has a mild effect on serum osmolality (Singhi 2008).

Hypertonic saline and sodium lactate appear to have direct osmotic actions on cells and they do not cause diuresis. These drugs may therefore be better than mannitol in reducing ICP (Ichai 2009). Osmotic diuretics such as mannitol and sorbitol could potentially also have a clinical benefit in meningitis through reduction in ICP but may risk volume depletion in the febrile patient. All osmotic therapies ideally require an intact blood brain barrier to exert their effects. Bacterial meningitis causes disruption of the barrier due to intense inflammation in the subarachnoid space and therefore it cannot be assumed that osmotic therapies would be beneficial. Table 2 gives details of all the properties of currently available osmotic therapies.

### Why it is important to do this review

To date, there have been a few placebo‐controlled studies using osmotic therapies in meningitis published in different settings in children and adults. A systematic review and meta‐analysis would help to decide if these studies have demonstrated clinical benefit either by improvement in mortality or long‐term neurological disabilities from the use of these treatments. This review aimed to encompass all types of osmotic therapies to investigate whether the principle of osmotic pressure change in the CNS is of benefit in people with meningitis and to demonstrate whether osmotic therapies should be recommended in principle, or if a particular therapy should be recommended in the treatment of acute bacterial meningitis.

## Objectives

To evaluate the effects of osmotic therapies added to antibiotics for acute bacterial meningitis in children and adults on mortality, deafness and neurological disability.

## Methods

### Criteria for considering studies for this review

#### Types of studies

Randomised controlled trials (RCTs).

#### Types of participants

Adults and children diagnosed with acute community‐acquired bacterial meningitis, as defined by the trial authors, on the basis of cerebrospinal fluid (CSF) culture, white cell count, biochemical composition and clinical presentation.

#### Types of interventions

**Intervention**: osmotic therapy, including at least one of the following: orally administered glycerol, intravenous (IV) hypertonic saline, sodium lactate and osmotic diuretics including IV mannitol and sorbitol.

**Control**: standard IV therapy or matched placebo.

All participants received broad‐spectrum intravenous antibiotic treatment.

#### Types of outcome measures

##### Primary outcomes

1. All‐cause mortality.

##### Secondary outcomes

1. Residual neurological deficit at the end of the follow‐up period, including focal neurological deficit, epilepsy and deafness. Deafness was defined as hearing loss greater than 40 decibels bilaterally.
2. Epilepsy/seizures.
3. Deafness (hearing loss greater than 40 decibels bilaterally).
4. Adverse effects.

### Search methods for identification of studies

#### Electronic searches

We searched the Cochrane Central Register of Controlled Trials (CENTRAL 2017, Issue 1), part of the *Cochrane Library,* www.thecochranelibrary.com (accessed 17 February 2017), which contains the Acute Respiratory Infections Group's Specialised Register, MEDLINE (Ovid) (1950 to 17 February 2017), Embase (Elsevier) (1974 to 17 February 2017), LILACS (BIREME) (1982 to 17 February 2017) and CINAHL (Ebsco) (1981 to 17 February 2017).

We used the search terms described in Appendix 1 to search MEDLINE and CENTRAL. We combined the MEDLINE search strategy with the Cochrane Highly Sensitive Search Strategy for identifying randomised trials in MEDLINE: sensitivity‐maximising version (2008 revision); Ovid format (Lefebvre 2011). We adapted the search strategy to search Embase (Appendix 2), CINAHL (Appendix 3) and LILACS (Appendix 4).

#### Searching other resources

We searched the following clinical trials registers on 21 February 2017.

1. ClinicalTrials.gov (www.clinical trials.gov) (Appendix 5).
2. World Health Organization International Clinical Trials Registry Portal (WHO ICTRP, www.who.int/ictrp/en/) (Appendix 6).

For previous versions of this review we also searched conference abstracts and contacted researchers in the field (to 12 December 2015).

### Data collection and analysis

#### Selection of studies

One author (EW) screened all search results (title and abstract) and selected relevant studies according to the review inclusion criteria. Two authors (EW, KA) screened all selected studies by reading the published full text to ensure each study met the inclusion criteria. The same two authors then agreed which studies were to be included in the review. We emailed trial authors to clarify duplication and study numbers.

#### Data extraction and management

Two review authors (EW, KA) independently extracted all data from the selected studies using a data extraction form. We discussed all trial data, which were then included only when the data matched those extracted by both review authors. We contacted one trial author regarding duplication and we excluded one study from the analysis as a result. No further discrepancies arose during data extraction. We entered data for analysis using RevMan 5.3 software (Review Manager 2014).

#### Assessment of risk of bias in included studies

The data extraction form included a 'Risk of bias' collection tool. Two review authors (EW, KA) independently judged the potential risk of bias for each included study as low, uncertain or high for the following parameters (Higgins 2011). Both review authors then discussed and agreed the final judgements. One review author (EW) synthesised these judgements into a standard 'Risk of bias' table for each study. See Characteristics of included studies.

1. Random sequence generation.
2. Allocation concealment.
3. Blinding.
4. Incomplete outcome data.
5. Selective reporting of outcome data.
6. Other identified areas of bias particular to that study (e.g. if the principal investigator was employed by the pharmaceutical company manufacturing the drug under investigation, or if the study is sponsored by a pharmaceutical company).

#### Measures of treatment effect

The primary outcome of this review was binary and the studies included were all RCTs, therefore we used the risk ratio (RR) as the most appropriate statistical tool to express the results of the treatment effect in a meta‐analysis. We displayed the results as forest plots.

All included studies had outcomes defined by the trial authors using standardised measurements. We counted hearing loss of greater than 40 decibels (dB) as significant where measured. If a formal neurological score was used to define neurological disability we used this. However, where only a description was given, we counted a described deficit that results in the participant not being able to work or attend school as significant. As the number of studies was small we were not able to analyse mortality by continental geographical area and resource setting as secondary outcomes, as planned in the protocol.

Due to the small number of studies retrieved, we were unable to group results for both primary and secondary outcomes by the follow‐up period: acute phase, less than three months since inclusion in the study and longer‐term up to one year of follow‐up.

#### Unit of analysis issues

We did not anticipate any cluster‐randomised trials on this topic. However, within the trials included, a four parallel‐arm design was employed. We separated data into groups comparing the intervention alone with placebo, and the intervention plus a second intervention with the second intervention alone. These results are expressed in Analysis 1.1 and Analysis 1.4.

#### Dealing with missing data

We found some relevant data to be missing from Kilpi 1995, Sankar 2007 and Molyneux 2014. We contacted the authors for clarification or additional data. Molyneux provided information and data; we did not receive responses from Kilpi or Sankar.

#### Assessment of heterogeneity

We intended to use the I² statistic and to explore explanations for heterogeneity by subgroup analysis as outlined in the protocol, but the data were insufficient.

#### Assessment of reporting biases

We assessed each study for reporting bias. Where it was suspected that selected results had been presented, we contacted the authors for clarification (see Dealing with missing data).

#### Data synthesis

We entered all extracted data into RevMan 5.3 (Review Manager 2014) and performed all analyses using this software. We expressed all results using forest plots. We used a fixed‐effect model for analysis and found minimal heterogeneity between the studies. We repeated the analyses using a random‐effects model where heterogeneity was detected. We present the results from the fixed‐effect model. Where disagreement in effect size was determined between the fixed‐effect and random‐effects models, we present data from both models.

##### GRADE and 'Summary of findings' table

We created Table 1 using the following outcomes: death, neurological disability, seizures, hearing loss and adverse effects*.* We used the five GRADE considerations (study limitations, consistency of effect, imprecision, indirectness and publication bias) to assess the quality of evidence as it related to the studies that contributed data to the meta‐analyses for the prespecified outcomes (Atkins 2004). We used the methods and recommendations described in Section 8.5 and Chapter 12 of the *Cochrane Handbook for Systematic Reviews of Interventions* (Higgins 2011) using GRADEpro GDT software (GRADEpro GDT 2014). We justified all decisions to downgrade or upgrade the quality of studies in footnotes, and made comments to aid readers' understanding of the review where necessary.

## Results

### Description of studies

#### Results of the search

We obtained 31 records from the 2017 update search; two duplicates were excluded. We assessed 29 records and could exclude 19 titles and abstracts. We obtained five full texts and excluded four. We included one new study in this update (Molyneux 2014).

We screened a total of 752 abstracts following the initial search in November 2010. Further records were screened following update searches in November 2012 (35 records from electronic databases), November 2014 (24 records) and February 2017 (24 records from electronic databases and five records from trials databases). This resulted in 840 screened abstracts in total over the history of this review including updates. See Figure 1.

**1:**
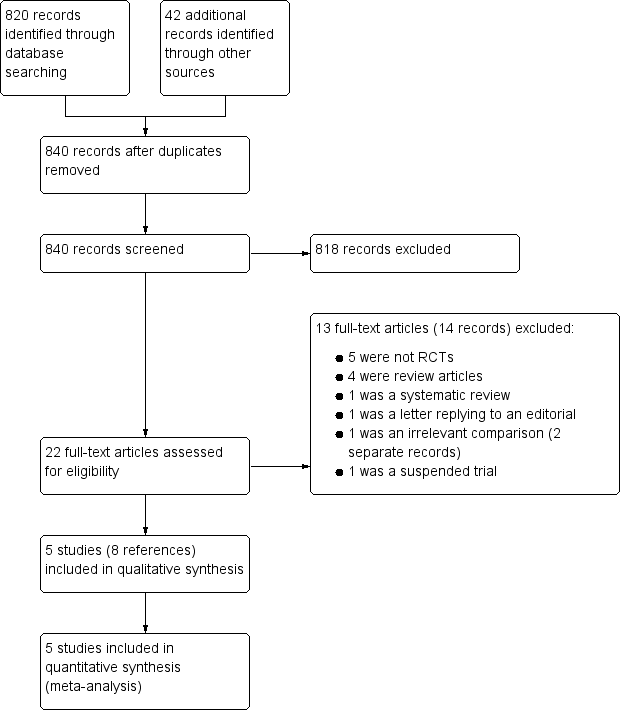
Study screening flow diagram

#### Included studies

Five trials, published in eight trial reports, with a total of 1451 participants met the inclusion criteria (Ajdukiewicz 2011; Kilpi 1995; Molyneux 2014; Peltola 2007; Sankar 2007). Molyneux 2014 was added at this update and included 181 participants. We extracted no data from a companion paper to Sankar 2007; it reported osmolarity data for a subset. We similarly extracted no data from a companion paper to Peltola 2007; it reported on deafness in more detail.

All included studies tested glycerol compared to matched placebo, with some studies including a dexamethasone arm and one study an acetaminophen (paracetamol) arm.

##### Study funding sources

Four studies were funded by research foundations (Ajdukiewicz 2011; Kilpi 1995; Molyneux 2014; Peltola 2007) and of these, two studies were also partially funded by the pharmaceutical industry (Kilpi 1995; Peltola 2007). One study reported no funding (Sankar 2007).

##### Participants

Four trials were conducted in children aged under 16 years (Kilpi 1995; Molyneux 2014; Peltola 2007; Sankar 2007) and one in adults and adolescents aged over 14 years (Ajdukiewicz 2011).

##### Interventions

All included studies used oral glycerol as the primary intervention. The potential mechanism of action of glycerol is detailed in Table 2. The four trials in children evaluated glycerol alone, dexamethasone alone, glycerol combined with dexamethasone and glycerol combined with paracetamol. These studies used intravenous (IV) placebo to 'blind' the dexamethasone treatment group. No placebo for oral glycerol was used in Kilpi 1995 and Sankar 2007. Peltola 2007 and Molyneux 2014 used oral carboxymethylcellulose as a placebo for glycerol.

The adult study used 50% dextrose as an oral placebo agent to compare to glycerol diluted in water or 50% dextrose (Ajdukiewicz 2011).

##### Location

Kilpi 1995 took place in Finland, Peltola 2007 in South America (multiple sites), Sankar 2007 in India and both Ajdukiewicz 2011 and Molyneux 2014 in Malawi.

##### Outcomes

Death was the primary outcome in all included studies.

In Peltola 2007, we noted different results in tables 2 and 3. As there appeared to be exclusions in table 3, we used the data from table 2, which appeared to be intention‐to‐treat.

#### Excluded studies

We excluded 13 studies (14 records). We found that 11 studies, which each used or mentioned the use of osmotic therapies, were not randomised controlled trials (RCTs) and these were excluded. Reasons for exclusion were as follows:

- five studies were not randomised trials;
- four were review articles;
- one was a systematic review (we screened the reference list and found no new studies to include in our review); and
- one was a letter replying to an editorial comment.

We also excluded one study that included children with acute central nervous system infections and raised intracranial pressure (ICP) randomised to receive cerebral perfusion pressure‐targeted therapy or intracranial pressure‐targeted therapy (Kumar 2014); and one study that was a registered trial record (CTRI/2015/04/005668). The trial registry stated that it had been suspended and this was confirmed with the trialists. See Figure 1 for a flow diagram of the study selection process.

##### Studies awaiting classification

There are currently no studies awaiting classification.

##### Ongoing studies

We did not identify any ongoing studies.

### Risk of bias in included studies

Risk of bias was mostly low; 70% of our judgements were of low risk of bias (see Figure 2). See Figure 3 for our judgements for each risk of bias item for each included study.

**2:**
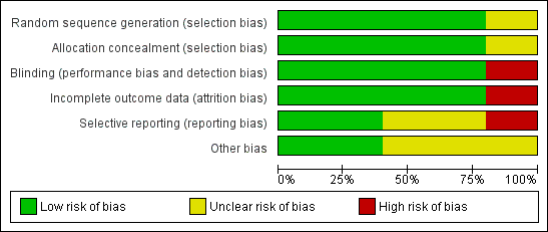
'Risk of bias' graph: review authors' judgements about each risk of bias item presented as percentages across all included studies

**3:**
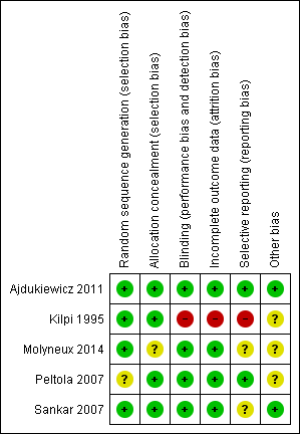
'Risk of bias' summary: review authors' judgements about each risk of bias item for each included study

#### Allocation

The risk of bias was low for random sequence generation across all studies. Allocation concealment was adequately described for all but one study (Molyneux 2014), which we judged at unclear risk of bias (Figure 2 and Figure 3). We judged Peltola 2007 at unclear risk of allocation bias due to changes in the protocol that occurred during the study, a change from two dexamethasone to one placebo to one dexamethasone to one placebo, as reported by a meta‐analysis of individual patient data testing dexamethasone compared to placebo for bacterial meningitis (van de Beek 2010).

#### Blinding

The risk of bias was low for blinding across four studies. We judged Kilpi 1995 at high risk of performance and detection bias, as no details of any concealment were given, so we assumed that the allocations were not blinded (Figure 2). The review authors requested clarification from the authors of Kilpi 1995 but no response has been received.

#### Incomplete outcome data

Four studies reported complete data and we judged them to have a low risk of attrition bias. Data on two participants were missing from Kilpi 1995 and we judged this study to have a high risk of attrition bias.

#### Selective reporting

We judged Ajdukiewicz 2011 and Peltola 2007 to have a low risk of reporting bias as all data appeared to be presented clearly and completely. Kilpi 1995 presented selected data as there was significant attrition bias, so we judged it to have a high risk of reporting bias. We judged Sankar 2007 to have an unclear risk of reporting bias as neither adverse effects nor time of stopping treatment were presented. Kilpi 1995 did not respond to our request for data on all enrolled trial participants.

#### Other potential sources of bias

No trials were sponsored by pharmaceutical companies, nor were the authors declared to have conflicts of interest. Peltola 2007 was partly funded by a pharmaceutical company, which supplied the dexamethasone for the trial but not the glycerol, so we did not judge this to have a significant bias effect on this analysis. Kilpi 1995 was also partially funded by a pharmaceutical company and we judged the risk of bias as unclear.

### Effects of interventions

See: Table 1

We included five trials, all evaluating glycerol. Four of the trials had four arms, which also compared glycerol plus dexamethasone with dexamethasone alone or glycerol plus paracetamol and paracetamol alone.

We carried out the initial analysis comparing participants who received glycerol or placebo only, or glycerol with paracetamol or placebo with paracetamol, labelled 'no steroids'. We carried out a subgroup analysis with the remaining trial participants who received either glycerol plus dexamethasone or dexamethasone plus placebo, labelled 'with steroids'. All trial participants received the antibiotic ceftriaxone, so no antibiotic subgroup analysis was necessary. Due to the small number of included studies, a subgroup analysis of paediatric data was not possible.

#### Primary outcome

##### All‐cause mortality

In the adult study, there were more deaths in the glycerol group and this led to the study being stopped by the data monitoring committee (risk ratio (RR) 1.30, 95% confidence interval (CI) 1.04 to 1.62) (Ajdukiewicz 2011). None of the other studies detected harm with glycerol and the meta‐analysis did not detect an effect on mortality (RR 1.08, 95% CI 0.90 to 1.30, 1272 participants, 5 trials, I² = 17%, Analysis 1.1, *moderate‐certainty evidence*). The stratified analysis found no significant difference whether dexamethasone was administered or not.

#### Secondary outcomes

##### 1. Residual neurological deficit at the end of the follow‐up period

Overall, a slight reduction (54/644 cases) in neurological disability was reported in the glycerol group compared with the placebo group (77/626) (RR 0.73, 95% CI 0.53 to 1.00, 1270 participants, 5 trials, I² = 50%, Analysis 1.2, *low‐certainty evidence*). The effect size was further reduced using the random‐effects model (RR 0.70, 95% CI 0.38 to 1.27). Little or no difference was detected in the subgroup of participants who received steroids (RR 0.82, 95% CI 0.38 to 1.77, 419 participants, 3 trials, I² = 25%).

##### 2. Epilepsy/seizures

Convulsions on admission and during treatment were reported in all studies but none reported data for persistent epileptic seizures post discharge. In the adult study, the risk of seizures was higher with glycerol (RR 1.62, 95% CI 1.18 to 2.23) (Ajdukiewicz 2011). However, this was not found in the other studies and the meta‐analysis did not detect a difference (RR 1.08, 95% CI 0.90 to 1.30; 1090 participants, 4 trials, I² = 54%, Analysis 1.3, *low‐certainty evidence*).

##### 3. Deafness

Fewer surviving participants given glycerol were reported as deaf at four to eight weeks of follow‐up compared to placebo (RR 0.64, 95% CI 0.44 to 0.93; 5 trials, 922 participants, I² = 7%, Analysis 1.4, *moderate‐certainty evidence*). Using the random‐effects model, the estimate of the effect size of glycerol on deafness was slightly lower (RR 0.67, 95% CI 0.44 to 1.01).

##### Adverse effects

Neither glycerol nor dexamethasone were associated with significant adverse effects in the included studies but systematic recording of adverse events was not reported. Only Ajdukiewicz 2011 reported on serious adverse events (SAEs). One SAE was reported each in glycerol and placebo arm participants, both considered possibly due to the study drug but the researchers reported that the most likely diagnosis for both participants (HIV‐positive adults in Malawi) was a major cerebrovascular event secondary to meningitis.

Common adverse effects were nausea and vomiting; there were also small numbers of cases of gastrointestinal bleeding.

###### Nausea, vomiting, diarrhoea

Two studies reported on nausea, vomiting or diarrhoea, with 221/426 events in the glycerol groups and 200/425 in the placebo groups. The meta‐analysis did not detect a difference (RR 1.09, 95% CI 0.81 to 1.47; 2 trials, 851 participants, I² = 79%, Analysis 1.5, *very low‐certainty evidence*) but heterogeneity was high. Peltola 2007, a study conducted with children in South America, reported more adverse events in the glycerol without steroids group (80/148) than in the placebo group (53/148) (RR 1.51, 95% CI 1.16 to 1.96; 296 participants).

Two studies reported results that could not be added to the meta‐analysis. Sankar 2007, a study conducted with children in India, reported that the incidence of vomiting in the glycerol and non‐glycerol groups was "similar", and Kilpi 1995, a trial with children conducted in Finland, reported a higher incidence of vomiting on days 2 and 3 in the glycerol and glycerol with steroid groups (day 2: 38%, day 3: 23%) than in the steroid and placebo groups (day 2: 14%, day 3: 4%) and that vomiting led to discontinuation of glycerol treatment in three cases.

###### Gastrointestinal bleeding

Overall, 10 cases (3%) of gastrointestinal bleeding were reported in each of the glycerol and placebo groups. The meta‐analysis did not detect a difference (RR 0.93, 95% CI 0.39 to 2.19; 3 trials, 607 participants, I² = 0%, Analysis 1.6, *moderate‐certainty evidence*).

## Discussion

### Summary of main results

We included five trials evaluating glycerol in acute bacterial meningitis. Other osmotic diuretics, such as mannitol and hypertonic saline, have not yet been tested.

Glycerol was tested in adults and children with acute bacterial meningitis in a variety of different clinical settings and in four of the five included trials, glycerol was evaluated in a complex trial design including dexamethasone or acetaminophen. The review and meta‐analysis did not detect an overall effect of glycerol on mortality from acute bacterial meningitis in children and adults. However, in the only trial in adults, glycerol was associated with increased mortality. We assessed the quality of the evidence using GRADE criteria as low (GRADEpro GDT 2014; Table 1).

The meta‐analysis of low‐quality evidence suggested that glycerol may reduce hearing loss (Table 1).

The small numbers seen overall in the studies in children were not sufficient to fully exclude the impact of dexamethasone, particularly on neurological disabilities and deafness in children, as this has been shown to be effective elsewhere (van de Beek 2010).

The overall number of study participants in this review was small and a significant degree of bias was found to be present in Kilpi 1995. Analysis was mainly weighted on Ajdukiewicz 2011 and Peltola 2007, two large studies that were both well conducted, but limited in their population demographics and follow‐up data. Data from Peltola 2007 have been subject to systematic reviews investigating the effect of dexamethasone, and some methodological concerns were raised regarding the randomisation schedule (van de Beek 2010). As a result we have assigned this study an unclear risk of allocation bias.

Each study was undertaken in a very different environment and the population for each has its own particular issues. The HIV prevalence in Ajdukiewicz 2011 was 83.5% and the impact of this on mortality and other outcomes has not been measured and may be significant. Ajdukiewicz 2011 and Molyneux 2014 were conducted in a severely resource‐limited environment in Malawi, with no access to advanced resuscitation or intensive care units (UNDP 2016). All other included studies were carried out in hospitals with intensive care units and paediatric specialist teams, which is not necessarily representative of most hospitals in low‐income countries. This may introduce a degree of confounding, particularly regarding lower mortality rates in children.

Peltola 2007 was conducted at multiple sites and excluded participants who had received parenteral antibiotics but not oral antibiotics before the first dose of glycerol or dexamethasone or both glycerol and dexamethasone. The authors of Peltola 2007 did not include these data in the analysis, so it is unclear if prior antibiotic treatment had an effect on outcomes, particularly deafness.

The doses and duration of glycerol used varied across the included studies, introducing further inconsistencies among studies (see Table 3). We were unable to control for this effect in the analysis, which may have introduced further heterogeneity (Brouwer 2011; Saez‐Llorens 2007). Prolonged use of osmotic agents, such as the four‐day courses of glycerol used in Ajdukiewicz 2011, have been suggested to be harmful. Peltola 2007 and Sankar 2007 both used two‐day courses due to this concern. However, most seizures and deaths in Ajdukiewicz 2011 occurred in the first two days, and therefore an association between mortality and glycerol duration is unlikely.

**2:** Comparison of included study interventions

**Name of study**	**Population**	**Intervention and dose**	**Control used**	**Treatment duration**	**Study arms**
Kilpi 1995	Children in Finland	Oral glycerol 4.5 g/kg max 180 g/24 h in 3 divided doses Dexamethasone (dex) 1.5 mg/kg max 60 mg/day	No oral placebo IV saline	3 days	4 arms: IV dexamethasone + glycerol, oral glycerol, IV dexamethasone, neither treatment
Sankar 2007	Children in India	Oral glycerol 1.5 g/kg 3 x daily Dexamethasone 0.15 mg/kg 3 x daily	Oral carboxymethylcellulose 2% IV saline	Not detailed	4 arms: placebo oral and IV, IV dexamethasone + oral glycerol, IV placebo + oral glycerol, IV dexamethasone + oral placebo
Peltola 2007	Children in South America	Oral glycerol 1.5 g/kg 3 x daily Dexamethasone 0.15 mg/kg 3 x daily	Oral carboxymethylcellulose 2% IV saline	2 days	4 arms: oral and IV placebo, IV dexamethasone + oral glycerol, IV placebo + oral glycerol, IV dexamethasone + oral placebo
Ajdukiewicz 2011	Adults in Malawi, Southern Africa	Oral glycerol 75 mg 4 x daily diluted in water or 50% dextrose solution	Oral 50% dextrose solution	4 days	Oral glycerol versus oral 50% dextrose
Molyneux 2014	Children in Malawi, Southern Africa	Oral glycerol 25 mL/dose (maximum dose) = 100 mL/24 hours. Acetaminophen 35 mg/kg 6‐hourly	Oral carboxymethylcellulose 2%	2 days	3 arms: oral glycerol and oral acetaminophen, oral placebo and glycerol, oral acetaminophen and oral placebo

IV: intravenous

Different agents were used as placebo comparators in the studies. Ajdukiewicz 2011 used 50% dextrose, Peltola 2007, Sankar 2007 and Molyneux 2014 used carboxymethylcellulose, and Kilpi 1995 did not use a placebo agent. It may be argued that the placebo agents used were not wholly inert and may exert an independent osmotic action. All trial authors designed control agents that had a similar taste and texture to glycerol for concealment purposes, and whether any of the substances used exerted an independent osmotic action is untested. However, the higher mortality reported by Ajdukiewicz 2011 in the glycerol group suggests that glycerol had an action beyond any osmotic effect exerted by the dextrose placebo, particularly as the glycerol was diluted in dextrose for some participants (Brouwer 2011).

The slight reduction in hearing loss observed suggests that glycerol may be acting to reduce oedema or improve cerebral blood flow in particular areas of the brain, either the nucleus or length of the vestibular‐cochlear nerve (which is encased in a bony canal). There is some evidence to suggest that glycerol is required for bacterial metabolic pathways in the central nervous system (CNS) (Mahdi 2012). Genetic susceptibility to hearing loss following meningitis has been suggested and the presence of glycerol may attenuate the production of free radicals that may affect CNS damage leading to hearing loss (van Well 2012). We selected greater than 40 dB as the cut‐off for hearing loss to capture all clinically significant deficits; the effect of glycerol on more severe hearing loss was not evaluated. Currently, there are no clear data showing the mechanistic effects of glycerol on either hearing or mortality in humans and more research is needed. Experimental animal work has shown no effect of glycerol in a bacterial meningitis model (Blaser 2010). The cause of increased mortality with glycerol in adults is unclear. Risk stratification of patients in that trial by disease severity showed that glycerol exerted harmful effects on those patients with low predicted risk of death on admission (Wall 2017). It is possible that increased mortality from glycerol in these patients with a more intact blood‐brain barrier may relate to enhanced virulence of pneumococci in the CNS in the presence of glycerol (Mahdi 2012), or harmful effects of osmotic shift across the blood‐brain barrier.

The use of dexamethasone did not have any impact on the outcomes studied when used with or without glycerol. Other larger reviews have found an impact of dexamethasone on the reduction of hearing loss in children with meningitis (van de Beek 2010). There were too few data available for analysis to inform a robust conclusion about the utility of dexamethasone for treatment of people with bacterial meningitis.

### Overall completeness and applicability of evidence

This is an update of a Cochrane Review that examines the evidence for the use of osmotic therapies in acute bacterial meningitis. To date, the evidence is incomplete and unequivocal findings for the use of glycerol for people with meningitis could not be derived. Data from further studies are required, particularly in children, to assess the impact of glycerol on meningitis‐induced hearing loss. There is no evidence testing any other osmotic therapy apart from glycerol for meningitis: data from clinical studies are required. The high‐quality evidence from Ajdukiewicz 2011 demonstrates harm from glycerol in adults with bacterial meningitis in Malawi and no further testing or clinical use of glycerol in adults is currently warranted.

### Quality of the evidence

We assessed the quality of evidence provided by this review using the GRADE methods (Table 1). We generally assessed the evidence as low‐ or very low‐quality, which indicates that further research is very likely to change the estimates of effect.

The main reasons for downgrading evidence quality were the small size of the trials, the low numbers of events and the substantial differences between locations, sizes and participant populations studied in the included studies. Much larger trials would be necessary to prove or exclude significant benefits or harms.

We also downgraded the evidence quality for mortality and seizures due to inconsistency. The only trial in adults was stopped early due to small but statistically significant harm (Ajdukiewicz 2011), while four trials in children did not demonstrate statistically significant effects.

### Potential biases in the review process

Dr Katherine Ajdukiewicz is an author of this Cochrane Review and was the principal investigator for one of the included studies. To minimise bias she did not extract any data from her study to include in the analysis or perform any of the analysis.

### Agreements and disagreements with other studies or reviews

There are no current systematic reviews examining glycerol or other osmotic agents for use in acute bacterial meningitis.

## Authors' conclusions

**Implications for practice**

There is no evidence to support the use of glycerol as adjunctive treatment for acute bacterial meningitis. Glycerol may have a small beneficial effect on reducing deafness in surviving children but further data are needed. Overall, the evidence quality is low.

**Implications for research**

Trials testing other osmotic interventions in acute bacterial meningitis may be considered, particularly in children.

## What's new

**Table:** 

**Date**	**Event**	**Description**
3 September 2017	New search has been performed	We updated our searches. We included one new trial (Molyneux 2014) and excluded six new trials (CTRI/2015/04/005668; Glimåker 2014; Kumar 2014; Molyneux 2015; Peltola 2013; Vaziri 2016). We added adverse events as an outcome and presented death and neurological disability separately. A new author joined the team to complete this update (Hanna Bergman).
17 February 2017	New citation required but conclusions have not changed	Our conclusions remain unchanged.

## Appendices

### Appendix 1. MEDLINE (Ovid) search strategy

1 exp Meningitis/ 2 meningit*.tw. 3 1 or 2 4 Osmosis/ 5 Osmotic Pressure/ 6 exp Diuretics, Osmotic/ 7 (osmos* or osmot* or osmol*).tw. 8 exp Sugar Alcohols/ 9 glycer*.tw,nm. 10 1,2,3‐propanetrio*.tw,nm. 11 mannitol*.tw,nm. 12 sorbit*.tw,nm. 13 Sodium Lactate/ 14 (sodium adj2 lactat*).tw,nm. 15 Saline Solution, Hypertonic/ 16 (hypertonic adj2 saline*).tw,nm. 17 or/4‐16 18 3 and 17

### Appendix 2. Embase (Elsevier) search strategy

#20 #16 AND #19 #19 #17 OR #18 #18 random*:ab,ti OR placebo*:ab,ti OR factorial*:ab,ti OR crossover*:ab,ti OR 'cross over':ab,ti OR 'cross‐over':ab,ti OR assign*:ab,ti OR allocat*:ab,ti OR volunteer*:ab,ti OR ((doubl* OR singl*) NEAR/2 (blind* OR mask*)):ab,ti #17 'randomized controlled trial'/exp OR 'single blind procedure'/exp OR 'double blind procedure'/exp OR 'crossover procedure'/exp #16 #3 AND #15 #15 #4 OR #5 OR #6 OR #7 OR #8 OR #9 OR #10 OR #11 OR #12 OR #13 OR #14 #14 (hypertonic NEAR/2 saline):ab,ti #13 'sodium chloride'/de #12 (lactat* NEAR/2 sodium):ab,ti #11 'lactate sodium'/de #10 sorbit*:ab,ti #9 mannitol*:ab,ti #8 '1,2,3‐propanetriol':ab,ti OR propanetrio*:ab,ti #7 glycer*:ab,ti #6 'sugar alcohol'/exp #5 osmotic*:ab,ti #4 'osmotic diuretic agent'/exp #3 #1 OR #2 #2 meningit*:ab,ti #1 'meningitis'/exp

### Appendix 3. CINAHL (Ebsco) search strategy

S12 S3 and S11 S11 S4 or S5 or S6 or S7 or S8 or S9 or S10 S10 TI hypertonic N2 saline or AB hypertonic N2 saline S9 (MH "Saline Solution, Hypertonic") S8 TI sodium N2 lactat* or AB sodium N2 lactat* S7 TI ( glycerol* or 1,2,3‐propanetriol or propanetriol* or mannitol* or sorbit* ) or AB ( glycerol* or 1,2,3‐propanetriol or propanetriol* or mannitol* or sorbit* ) S6 AB sugar alcohol* or TI sugar alcohol* S5 (MH "Sugar Alcohols+") S4 TI osmotic* or AB osmotic* S3 S1 or S2 S2 TI meningit* or AB meningit* S1 (MH "Meningitis+")

### Appendix 4. LILACS (BIREME) search strategy

> Search > (MH:Meningitis OR Meningite OR MH:C10.228.228.507$ OR C10.228.566$ OR MH:C01 252.200$ OR C10.228.228.180.500$ OR meningit$) AND (MH:"Diuretics, Osmotic" OR "Diuréticos Osmóticos" OR "Diuréticos Osmóticos" OR "Osmotic Diuretics" OR MH:D27.505.696.560.500.453$ OR osmot$ OR osmos$ OR osmol$ OR MH:"Sugar Alcohols" OR "Alcoholes del Azúcar" OR "Álcoois de Açúcar" OR MH:D02.033.800$ OR MH:D09.853$ OR "sugar alcohols" OR glycer$ OR "1,2,3‐propanetriol" OR mannitol$ OR sorbit$ OR MH:"Sodium Lactate" OR "Lactato de Sodio" OR "Lactato de Sódio" OR MH:D02.241.511.459.500$ OR "sodium lactate" OR MH:"Saline Solution, Hypertonic" OR "Solución Salina Hipertónica" OR "Solução Salina Hipertônica" OR "Hypertonic Saline Solution" OR "Sodium Chloride Solution, Hypertonic" OR "Hypertonic Solution, Saline" OR "Solución Hipertónica de Cloruro de Sodio" OR "Solução Hipertônica de Cloreto de Sódio" OR "hypertonic saline") > clinical_trials

### Appendix 5. ClinicalTrials.gov search strategy

Search terms: (meningitis OR meningitides) AND (osmosis or osmoses or osmotic or osmolarity or glycerol OR glycerine OR glycerin OR or 1,2,3‐propanetriol or propanetriol or mannitol or sorbitol or sodium lactate or (hypertonic AND saline))

### Appendix 6. WHO ICTRP search strategy

Search terms: meningit* AND osmos* or meningit* AND osmot* OR meningit* AND osmol* or meningit* AND glycer* OR meningit* AND 1,2,3‐propanetrio* or meningit* AND mannitol* or meningit* AND sorbit* or meningit* AND sodium lactate or meningit* AND hypertonic AND saline*

## Data and analyses

**Comparison 1:** Glycerol versus no osmotic diuretic

Outcome or subgroup title	No. of studies	No. of participants	Statistical method	Effect size
1 Death	5	1272	Risk Ratio (M‐H, Fixed, 95% CI)	1.08 [0.90, 1.30]
1.1 No steroids	5	853	Risk Ratio (M‐H, Fixed, 95% CI)	1.10 [0.90, 1.33]
1.2 With steroids	3	419	Risk Ratio (M‐H, Fixed, 95% CI)	1.02 [0.60, 1.74]
2 Neurological disability	5	1270	Risk Ratio (M‐H, Fixed, 95% CI)	0.73 [0.53, 1.00]
2.1 No steroids	5	851	Risk Ratio (M‐H, Fixed, 95% CI)	0.71 [0.49, 1.01]
2.2 With steroids	3	419	Risk Ratio (M‐H, Fixed, 95% CI)	0.82 [0.38, 1.77]
3 Seizures	4	1090	Risk Ratio (M‐H, Fixed, 95% CI)	1.08 [0.90, 1.30]
3.1 No steroids	4	755	Risk Ratio (M‐H, Fixed, 95% CI)	1.15 [0.92, 1.44]
3.2 With steroids	2	335	Risk Ratio (M‐H, Fixed, 95% CI)	0.96 [0.70, 1.33]
4 Hearing loss	5	922	Risk Ratio (M‐H, Fixed, 95% CI)	0.64 [0.44, 0.93]
4.1 No steroids	4	572	Risk Ratio (M‐H, Fixed, 95% CI)	0.63 [0.41, 0.99]
4.2 With steroids	3	350	Risk Ratio (M‐H, Fixed, 95% CI)	0.66 [0.32, 1.35]
5 Adverse effects: nausea, vomiting, diarrhoea	2	851	Risk Ratio (M‐H, Random, 95% CI)	1.09 [0.81, 1.47]
5.1 No steroids	2	546	Risk Ratio (M‐H, Random, 95% CI)	1.22 [0.81, 1.83]
5.2 With steroids	1	305	Risk Ratio (M‐H, Random, 95% CI)	0.86 [0.66, 1.13]
6 Adverse effects: gastrointestinal bleeding	3	607	Risk Ratio (M‐H, Random, 95% CI)	0.93 [0.39, 2.19]
6.1 No steroids	3	296	Risk Ratio (M‐H, Random, 95% CI)	0.39 [0.06, 2.60]
6.2 With steroids	3	311	Risk Ratio (M‐H, Random, 95% CI)	1.16 [0.44, 3.04]

## Characteristics of studies

### Characteristics of included studies [ordered by study ID]

**Table:** Ajdukiewicz 2011

Methods	Randomised controlled trial	
Participants	Adults with bacterial meningitis (clinical suspicion of meningitis plus CSF evidence of infection: > 100 white cells/mm³, predominately neutrophils, a positive gram stain or cloudy CSF)	
Interventions	Oral glycerol 75 mg in 135 mL Oral glucose 50% solution 135 mL	
Outcomes	Primary outcome: mortality Secondary outcomes: epilepsy, deafness, residual neurological deficit at day 40	
Notes	Source of funding: the Meningitis Research Foundation Placebo is potentially not completely inactive and 50% glucose may exert a neurological effect in meningitis	
***Risk of bias***		
**Bias**	**Authors' judgement**	**Support for judgement**
Random sequence generation (selection bias)	Low risk	"A randomisation number list in blocks of 12 was produced by an independent statistician using Stata version 9.0"
Allocation concealment (selection bias)	Low risk	"Numbers and allocation were placed into sealed envelopes. Envelopes were opened sequentially by an independent person not involved in the clinical care or assessment of trial participants"
Blinding (performance bias and detection bias) All outcomes	Low risk	"Triple blinded"
Incomplete outcome data (attrition bias) All outcomes	Low risk	Intention‐to‐treat analysis; all participants included in the analysis
Selective reporting (reporting bias)	Low risk	None apparent
Other bias	Low risk	No other biases apparent

**Table:** Kilpi 1995

Methods	Randomised controlled trial with 4 arms	
Participants	Children from 3 months to 15 years of age with bacterial meningitis (CSF culture positive; CSF leucocytes > 100/mm²; positive blood culture in a child with signs and symptoms of bacterial meningitis)	
Interventions	Glycerol 4.5 g/kg to a maximum 180 g/day divided into 3 doses/24 hours. Increased by 50% for dose 1 and decreased by 50% for dose 2. No details of placebo given. Treatment given for 3 days Dexamethasone 1.5 mg/kg once daily IV divided into 3 doses/24 hours. 50% dose adjustments as per glycerol also used. Treatment given for 3 days 4 groups used, glycerol, glycerol + dexamethasone, dexamethasone and "neither"	
Outcomes	Primary outcome: mortality Secondary outcomes: epilepsy, deafness, residual neurological deficit	
Notes	Source of funding: the Arvo and Lea Ylppö Foundation, Helsinki, Finland, and Roche Oy, Helsinki, Finland No details given of whether any placebo agent was used	
***Risk of bias***		
**Bias**	**Authors' judgement**	**Support for judgement**
Random sequence generation (selection bias)	Low risk	"A computer generated list of random therapy assignments was kept at the children's hospital"
Allocation concealment (selection bias)	Low risk	"The next adjunctive treatment regimen was obtainable by telephone 24 hours a day" It was not clear if this person giving the assignments was part of the study team or independent
Blinding (performance bias and detection bias) All outcomes	High risk	No details of blinding were given, so we assumed the study was unblinded
Incomplete outcome data (attrition bias) All outcomes	High risk	134 children enrolled, 12 excluded, 122 in the final series but only 120 analysed. Details of the missing data were not present in the text
Selective reporting (reporting bias)	High risk	No details of the missing data given, so it is not clear if selective cases are presented
Other bias	Unclear risk	Groups not completely matched: more females in the dexamethasone group and increased meningitis due to *S pneumoniae* in the control group

**Table:** Molyneux 2014

Methods	Randomised controlled trial with 4 arms	
Participants	Children aged 2 months or older with bacterial meningitis (CSF culture positive; CSF leucocytes ≥ 100/mm² with positive blood culture; CSF ≥ 100 leukocytes with signs and symptoms of bacterial meningitis)	
Interventions	1. Glycerol + paracetamol 2. Glycerol 3. Paracetamol 4. Placebo All placebo‐controlled: carboxymethylcellulose (placebo for glycerol) and cocoa butter base suppository (placebo for paracetamol) Doses: glycerol 6 g/kg/day in 4 daily doses (maximum 2.5 mg/dose) for 2 days Acetaminophen rectal suppository 35 mg/kg first dose followed by 20 mg/kg 6‐hourly for 42 hours	
Outcomes	Primary outcome: Survival to 6 months post discharge with no sequelae Secondary outcomes: Sequelae that affect daily life (e.g. hemiplegia, deafness, blindness, seizures, global developmental delay) at 6 months Severe incapacitating sequelae Death	
Notes	Source of funding: the Academy of Finland In the trial registration from 2008, the primary outcomes were: death, severe neurological sequelae and hearing loss; secondary outcomes were: audiological or neurological sequelae	
***Risk of bias***		
**Bias**	**Authors' judgement**	**Support for judgement**
Random sequence generation (selection bias)	Low risk	"...randomisation was computer generated in permuted blocks of 12"
Allocation concealment (selection bias)	Unclear risk	No information
Blinding (performance bias and detection bias) All outcomes	Low risk	No report in trial. Email from author that the trial was "double blind"
Incomplete outcome data (attrition bias) All outcomes	Low risk	Intention‐to‐treat analysis; all participants included in the analysis
Selective reporting (reporting bias)	Unclear risk	None apparent. Some differences between trial report and trial registration
Other bias	Unclear risk	No detailed baseline characteristics: "baseline data for the 4 groups were similar except more children had received antibiotics in the paracetamol + glycerol group"

**Table:** Peltola 2007

Methods	Randomised controlled trial with 4 arms, multicentre in South America	
Participants	Children aged 2 months to 16 years with bacterial meningitis (CSF culture positive, "characteristic CSF findings" with a positive blood culture or CSF positive with latex antigen test; symptoms and signs of bacterial meningitis with at least 3 of the following: CSF white cell count > 1000 cells/mm³, CSF glucose < 40 mg/dL, CSF protein > 40 mg/dL, blood white cell count >15,000 cells/mm³	
Interventions	Glycerol 1.5 g/kg in an 85% solution divided into 3 doses/24 hours. Treatment given for 2 days Placebo: saline plus carboxy methylcellulose. Doses and volumes of placebo not given in the paper Dexamethasone 0.15 mg/kg once daily IV divided into 3 doses/24 hours. Treatment given for 2 days 4 groups: glycerol + placebo, glycerol + dexamethasone, dexamethasone + placebo and placebo + placebo	
Outcomes	Primary mortality. No secondary mortality at the end of follow‐up given Secondary outcomes: epilepsy, deafness and residual neurological deficit	
Notes	Source of funding: GlaxoSmithKline, Alfred Kordelin, Päivikki and Sakari Sohlberg, and Sigfrid Jusélius Funds. Farmacia Ahumada donated glycerol and both placebo preparations. Laboratorio de Chile partly donated ceftriaxone.	
***Risk of bias***		
**Bias**	**Authors' judgement**	**Support for judgement**
Random sequence generation (selection bias)	Unclear risk	"Stratified block randomisation took place in blocks of 20"
Allocation concealment (selection bias)	Low risk	"All treatment kits were packaged according to the randomisation lists in Santiago, Chile. Saline and carboxymethylcellulose were the placebo preparations for dexamethasone and glycerol, respectively. The agents were provided in identical ampoules or bottles and were labelled only with a study code"
Blinding (performance bias and detection bias) All outcomes	Low risk	"All treatment kits were packaged according to the randomisation lists in Santiago, Chile. Saline and carboxymethylcellulose were the placebo preparations for dexamethasone and glycerol, respectively. The agents were provided in identical ampoules or bottles and were labelled only with a study code"
Incomplete outcome data (attrition bias) All outcomes	Low risk	None identified
Selective reporting (reporting bias)	Low risk	No missing data identified
Other bias	Unclear risk	Drugs were supplied by GlaxoSmithKline (GSK) and Farmacia Ahumada. GSK partially funded the study

**Table:** Sankar 2007

Methods	Randomised controlled trial. Single centre	
Participants	Children aged 2 months to 12 years with bacterial meningitis (positive CSF culture or CSF latex agglutination positive, or CSF cytology with a suggestive biochemical profile with fever and signs of CNS involvement)	
Interventions	Glycerol 1.5 g/kg IV or orally 6‐hourly. Placebo carboxymethyl cellulose 2% solution IV. Total dose of placebo not given just documented "matched". Dexamethasone 0.15 mg/kg 6‐hourly. Duration of treatment not reported	
Outcomes	Primary mortality. No secondary mortality at the end of follow‐up given Secondary outcomes: epilepsy, deafness and residual neurological deficit	
Notes	Source of funding: reported as "Nil" This study was published twice, with a preliminary analysis of the osmotic effects published as Singhi 2008	
***Risk of bias***		
**Bias**	**Authors' judgement**	**Support for judgement**
Random sequence generation (selection bias)	Low risk	Randomisation list prepared with a simple random numbers table
Allocation concealment (selection bias)	Low risk	Serially numbered, sealed packets prepared, kept readily available
Blinding (performance bias and detection bias) All outcomes	Low risk	Clinicians and participants blinded. It was not clear from the text if the investigators were fully blinded but the packets were prepared by a separate person from the investigating team
Incomplete outcome data (attrition bias) All outcomes	Low risk	Outcome data were complete
Selective reporting (reporting bias)	Unclear risk	No data were reported for important outcomes: adverse events and time for stopping treatment
Other bias	Low risk	No other biases apparent

CNS ‐ central nervous system; CSF ‐ cerebrospinal fluid; IV ‐ intravenous

### Characteristics of excluded studies [ordered by study ID]

**Table:**

Study	Reason for exclusion
Almirante 1995	Case series of mannitol used for bacterial meningitis. No randomisation or placebo use documented
CTRI/2015/04/005668	RCT of newborns with bacterial meningitis receiving oral glycerol versus standard treatment. Eligible for inclusion, but the trial was suspended. This was confirmed by the trialists
Glimåker 2014	Not a RCT: retrospectively identified controls. Osmotherapy (hypertonic saline) was one of the interventions
Herson 1977	Not a RCT. Glycerol use discussed
Kumar 2014	Open‐label RCT of children with raised intracranial pressure due to acute CNS infections, including meningitis receiving fluid and vasoactive therapy to maintain cerebral perfusion pressure above 60 mm Hg versus hyperventilation and osmotherapy to maintain intercranial pressure below 20 mm Hg
Molyneux 2015	Review article. Glycerol use discussed
Pecco 1991	Literature review and documented personal experience of the use of mannitol in meningitis
Pelegrin 2012	Retrospective cohort study examining patients with bacterial meningitis 1987 to 2009 who were treated with dexamethasone, mannitol and phenytoin. No data were collected prospectively and participants were not randomised to receive any of the interventions
Peltola 2013	Review article. Glycerol use discussed
Singhi 2004	Review article. Not a RCT
Singhi 2007	Letter in response to the journal editorial summary of Peltola 2007
Urciuoli 1963	Mannitol tested for neurosurgical infections and not acute bacterial meningitis. Not a RCT
Vaziri 2016	Systematic review. Glycerol use discussed

CNS ‐ central nervous system; RCT ‐ randomised controlled trial

## Differences between protocol and review

In the 2017 update, we presented death and disability separately. In the earlier version of this review, this was a composite outcome. We believe this provides greater clarity for patients and clinicians.
